# Role of a thrombin generation assay in the prediction of infection severity

**DOI:** 10.1038/s41598-021-86915-7

**Published:** 2021-04-09

**Authors:** Boaz Elad, Gilat Avraham, Naama Schwartz, Adi Elias, Mazen Elias

**Affiliations:** 1 Department of Cardiology, Rambam Health Care Campus, Haifa, Israel; 2 Department of Internal Medicine C, Emek Medical Center, Afula, Israel; 3 School of Public Health, University of Haifa, Haifa, Israel; 4 Department of Internal Medicine B, Rambam Health Care Campus, Haifa, Israel; 5 Rapaport Faculty of Medicine, Technion Institute of Technology, Haifa, Israel

**Keywords:** Microbiology, Haematological diseases

## Abstract

Thrombin plays a central role in sepsis pathophysiology. The correlation of thrombin generation (TG) assays with infection severity and prognosis, and whether it can be used as a clinical tool, have been poorly explored and are the subjects of our research. We recruited 130 patients with systemic infection between 2016 and 2019. Patients were divided according to infection severity by using the sequential organ failure assessment (SOFA) and quickSOFA (qSOFA) scores. The hemostatic state was analyzed by Calibrated Automated Thrombogram. The primary end points were TG values and the secondary end point was in-hospital mortality. Patients with qSOFA ≥ 2 had a longer lag time (5.6 vs. 4.6 min) and time to peak (8 vs. 6.9 min) than those with lower scores (p = 0.014 and 0.01, respectively). SOFA ≥ 2 had a longer lag time (5.2 vs. 4.3 min), time to peak (7.5 vs. 6.7 min) and lower endogenous thrombin potential (ETP) (1834 vs. 2015 nM*min), p = 0.008, 0.019, and 0.048, respectively. Patients who died (11) had lower ETP (1648 vs. 1928 nM*min) and peak height (284 vs. 345 nM), p = 0.034 and 0.012, respectively. In conclusion TG assays may be a valuable tool in predicting infection severity and prognosis.

## Introduction

Sepsis is a leading cause of morbidity and mortality worldwide^1^. Severity of sepsis is assessed by scoring systems. The predominant score in current use is the sequential organ failure assessment (SOFA) score, which grades the dysfunction of six organ systems using laboratory variables and clinical signs and symptoms. High SOFA scores are associated with increased mortality. SOFA scores ≥ 2 reflect an overall mortality risk of approximately 10% and identified a 2- to 25-fold increased risk of dying compared with patients with a SOFA score < 2^2^. In general hospital ward settings, patients were found to have poor outcomes if they demonstrated a high bedside clinical score, named quickSOFA (qSOFA)^2^. Sepsis is almost invariably associated with coagulation abnormalities that range from mild changes that can only be identified by highly sensitive assays, to fulminant disseminated intravascular coagulation (DIC)^3^. Thrombin formation is central to the activation of coagulation in sepsis and exerting numerous cellular effects, playing a major role in the pathophysiology of sepsis ^4^. Therefore, in patients with sepsis, thrombin seems to be a valuable research topic. Unfortunately, conventional coagulation tests (i.e. prothrombin time, activated partial tromboplastin time) are not a reliable tool for the assessment of thrombotic or hemorrhagic phenotypes. It seems that the measurement of an individual’s capacity to generate thrombin is potentially more useful than conventional coagulation tests^5^. Calibrated automated thrombogram (CAT), a fluorogenic thrombin generation (TG) assay, can be used to measure TG in multiple samples simultaneously. The increased use of TG assays raises questions regarding their role in clinical practice. Whether TG is correlated with infection severity and prognosis, and whether it can be used as a clinical tool, have been poorly explored thus far and are the main topics of our present research.

## Methods

### Study design

In this prospective, single-center study we recruited patients with systemic infection and suspected sepsis. Within the first 24 h after the diagnosis of systemic infection, blood tests and clinical measurements were performed for evaluation of infection severity by calculating SOFA and qSOFA scores; the hemostatic state was analyzed by CAT, a TG assay. Demographics and comorbidities were meticulously collected from electronics records. This study was performed in line with the principles of the Declaration of Helsinki. Approval was granted by the ethics committee of Emek medical center, reference number EMC131-16.

### Study population

Eligible patients included patients ≥ 18 years old with newly diagnosed systemic infectious disease (in the preceding 24 h) and suspected sepsis who were willing to sign an informed consent form. Patients who could not sign were evaluated and approved for recruitment by an unbiased physician not associated with the research. Patients with systemic infectious disease were suspected of sepsis if they had increased inflammatory markers (white blood cell count, C reactive protein), compromised hemodynamics measurements (systolic BP < 90 or diastolic BP < 60, tachycardia > 90 beats/minute, fever > 38 or < 36 °C, respiratory rate > 20, decreased mental state) or instant positive bacteremia. Patients were recruited from internal wards, the intensive care unit and emergency room department of the Emek Medical Center, in Afula, Israel. Exclusion criteria included patients known to have thrombophilia, coagulopathy, active oncological disease, those known to be current users of anticoagulation medication and pregnancy.

### Thrombin generation

TG evaluation was conducted with the use of a CAT assay (Thrombinoscope B.V, Maastricht, the Netherlands) for analysis of platelet-poor plasma (PPP). Six milliliters of blood were collected in two tubes containing sodium citrate (3 ml, 9NC Coagulation sodium citrate 3.2%).

**Figure 1:**
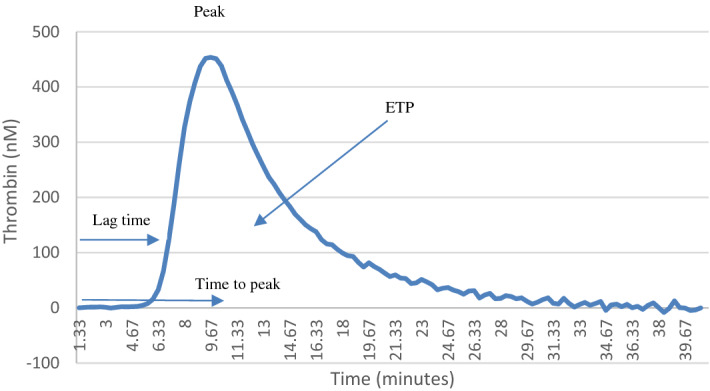
Calibrated Automated Thrombogram output in suspected sepsis.

To obtain PPP the samples were centrifuged twice, first for 15 min at 2500 revolutions per minute (RPM) and then for 10 min at 2000 RPM. The plasma was stored at − 70 °C for later analysis. According to the manufacturer's instructions, measurements were conducted on 80 μl of PPP triggered by 20 μl PPP-reagent (4 μmol phospholipid and 5 pmol tissue factor). Measurements were calibrated with 20 μl Thrombin Calibrator, Fluorogenic substrate (20 μl) was added to sample. The thrombogram generated four parameters: lag time (in minutes), time to peak (TTpeak) (in minutes), peak height (nM) and endogenous thrombin potential (ETP) (in nM*minute), representing the area under the TG curve^6,7^. An example of CAT assay output is shown in Fig. 1.

### Infection severity scores

The SOFA score grades the dysfunction of six organ systems using laboratory variables (partial pressure of oxygen, fraction of inspired oxygen, platelets, bilirubin, creatinine) and clinical signs and symptoms (mechanical ventilation, Glasgow coma score, mean arterial pressure, use of vasoactive agents, urine output). The qSOFA score includes the following clinical criteria: respiratory rate ≥ 22/min, altered mentation, systolic blood pressure (BP) ≤ 100 mm Hg. For each patient, a qSOFA score (values between 0 and 3) and a SOFA score (0–24) were calculated (www.medcalc.com). Laboratory variables including complete blood count, chemistry tests, arterial blood gases and blood cultures were taken and analyzed by the Emek Medical Center laboratory within the first 24 h of infectious disease diagnosis. Clinical signs and symptoms were collected from electronic records.

### End points

Primary: TG parameters-lag time, time to peak, peak height and ETP.

Secondary: in-hospital mortality.

### Statistical analysis

The study population was divided into two groups by their SOFA and qSOFA scores (high vs. low). Cut off values were chosen reflecting high vs. low mortality risk^2^. Based on former research^8^, to see a mean difference in ETP of 300 nM*min between the low- and high-score groups with standard deviation (SD) of 480 for both groups and a 1:4 group ratio, we needed to recruit a total of 130 patients for a power of 80% and alpha value of 5% (two-sided test). Categorical variables (i.e., gender) were represented by frequency and percentage, and continuous variables (i.e., ETP) were represented by standard distribution measurement (i.e., mean, SD). Low and high SOFA and qSOFA scores were compared to CAT values by using a t-test. For categorical variables, a chi-square test or Fisher test were used. The associations between categorical or continuous variables and each of the CAT values were examined with multivariate general linear model. The multivariate analysis was performed with the stepwise algorithm (the final model included only variables with P < 0.05). In addition, the correlation between the qSOFA and SOFA scores (as an ordinal variable) and CAT values was tested by using Spearman correlation. The accuracy of SOFA, qSOFA and CAT values were assessed by comparing receiver operating characteristic (ROC) curves using the test proposed by DeLong et al.^9^. Statistical analysis was conducted by SAS 9.4 software, and statistical significance was considered when p < 0.05.

## Results

Between 2016 and 2019, one hundred and thirty patients with systemic infection were recruited. Eight of these patients did not have a fully calculated qSOFA or SOFA scores, or CAT values due to technical problems and were dropped out. Demographics and comorbidities varied; patients with higher SOFA/qSOFA scores were older and sicker (with higher rates of stroke, hypertension, and heart failure). Higher SOFA scores were noted in men and in patients with chronic renal failure (Table 1).

**Table 1:** Demography and medical history.

	QSOFA			SOFA		
	0–1	2–3	P-value	0–1	2 +	P-value
	N = 93	N = 29		N = 48	N = 66	
Age	61.5 (19.3)	73.1 (18.1)	0.002	57.5 (22)	69.2 (15.9)	0.0025
Gender; Male	47 (50.54%)	15 (51.72%)	0.9111	20 (41.67%)	39 (59.09%)	0.066
BMI	28.1 (8.2)	28.4 (10)	0.7982	27.6 (8.8)	29 (8.6)	0.4032
Obesity	29 (31.87%)	7 (26.92%)	0.6299	11 (22.92%)	23 (37.7%)	0.098
INR	1.1 (0.2)	1.2 (0.2)	0.1924	1.1 (0.2)	1.2 (0.2)	0.0036
IHD	16 (17.2%)	8 (27.59%)	0.2195	7 (14.58%)	17 (25.76%)	0.1485
CVA/TIA	12 (12.9%)	15 (51.72%)	< .0001	4 (8.33%)	23 (34.85%)	0.001
COPD	19 (20.43%)	3 (10.34%)	0.2174	7 (14.58%)	12 (18.18%)	0.6107
DM	43 (46.24%)	17 (58.62%)	0.2441	19 (39.58%)	37 (56.06%)	0.0823
HTN	49 (52.69%)	23 (79.31%)	0.0109	19 (39.58%)	48 (72.73%)	0.0004
CHF	12 (12.9%)	9 (31.03%)	0.0448	4 (8.33%)	15 (22.73%)	0.0417
Liver disease	2 (2.15%)	0 (0%)	> 0.99	2 (4.17%)	0 (0%)	0.1751
CKD	14 (15.05%)	7 (25%)	0.2576	2 (4.17%)	19 (29.23%)	0.0007
**Diagnosis**						
Cellulitis	10 (10.75%)	0 (0%)		7 (14.58%)	3 (4.55%)	
Other	18 (19.35%)	2 (6.9%)		7 (14.58%)	12 (18.18%)	
Pneumonia	29 (31.18%)	17 (58.62%)		15 (31.25%)	25 (37.88%)	
UTI	36 (38.71%)	10 (34.48%)		19 (39.58%)	26 (39.39%)	
30-day mortality	1 (1.08%)	9 (31.03%)	< 0.0001	0 (0%)	9 (13.64%)	0.0098
In-hospital mortality	1 (1.08%)	10 (34.48%)	< 0.0001	0 (0%)	10 (15.15%)	0.0048

Categorical variables were analyzed with a chi-square test (or with Fisher’s exact test). Continuous variables are presented as the mean (SD). For continuous variables, the t-test was implemented.

*SOFA* sequential organ failure assessment, *qSOFA* quick sequential organ failure assessment, *BMI* body mass index, *DM* diabetes mellitus, *CKD* chronic kidney disease, *HTN* hypertension, *COPD* chronic obstructive pulmonary disease, *IHD* ischemic heart disease, *CHF* congestive heart failure, *CVA* cerebrovascular accident, *TIA*, transient ischemic attack, *UTI*, urinary tract infection, *M* male, *F* female.

Patients with severe infection, as determined by qSOFA ≥ 2, had a longer lag time (5.6 vs. 4.6 min) and TTpeak (8 vs. 6.9 min) than those with lower qSOFA scores, p = 0.014 and p = 0.01, respectively (Table 2).

**Table 2:** Thrombin generation and qSOFA/SOFA scores.

	QSOFA			SOFA		
	0–1	2–3	P-value	0–1	2 +	P-value
	N = 93	N = 29		N = 48	N = 66	
Lag time (min)	4.6 (1.3)	5.6 (2)	0.0143	4.3 (1.2)	5.2 (1.7)	0.0084
ETP (nmol*min)	1926.9 (477.1)	1828.5 (523.7)	0.3456	2015 (433.9)	1834.9 (504.4)	0.0486
Peak (nmol)	345.7 (70.8)	319.8 (76)	0.0927	353.4 (61.2)	331.8 (78.4)	0.1157
TTPeak (min)	6.9 (1.4)	8 (2.2)	0.0107	6.7 (1.4)	7.5 (1.8)	0.0197

Variables are presented as the mean (SD). For continuous variables, the t-test was implemented. SOFA, sequential organ failure assessment.

*qSOFA* quick sequential organ failure assessment, *Min* minute, *ETP* endogenous thrombin potential, *TTPeak* time to peak.

These significant differences were maintained after adjustment for risk factors and co-morbidities (Table 3).

**Table 3:** Thrombin generation and qSOFA/SOFA scores, adjustment for confounders.

Model adjustments	ETP		Peak height		Lag time		Time to peak	
	Β	*P*	Β	*P*	β	*P*	β	*P*
**qSOFA ≥ 2**								
Crude	− 0.086	0.346	− 0.153	0.093	0.284	0.001	0.294	0.001
*Multivariable model	− 0.016	0.878	− 0.140	0.180	0.310	0.003	0.344	0.001
**SOFA ≥ 2**								
Crude	− 0.185	0.049	− 0.148	0.116	0.275	0.003	0.218	0.020
*Multivariable model	− 0.125	0.245	− 0.071	0.504	0.244	0.023	0.180	0.099

β; the standardized regression coefficients, ETP (endogenous thrombin potential).

*Adjusted for age, Sex, BMI, diabetes mellitus, ischemic heart disease, stroke/TIA, COPD, hypertension, heart failure, chronic kidney disease.

Furthermore, the qSOFA score showed a positive correlation of 0.23 with lag time and 0.24 with TTpeak, p = 0.01 and 0.008, respectively. A negative correlation was observed between ETP and qSOFA (− 0.13) and peak height and qSOFA (− 0.17), p = 0.16 and 0.06 respectively (Table 4).

**Table 4:** Spearman correlation between thrombin generation and the SOFA/qSOFA ordinal scores.

	qSOFA (N = 122)		SOFA (N = 114)	
	Correlation	P-value	Correlation	P-value
Lag time	0.23	0.0106	0.30	0.0013
ETP	− 0.13	0.1698	− 0.16	0.0903
Peak	− 0.17	0.0646	− 0.12	0.1935
TTPeak	0.24	0.0088	0.27	0.004

*SOFA* sequential organ failure assessment, *qSOFA* quick sequential organ failure assessment, *Min* minute, *ETP* endogenous thrombin potential, *TTPeak* time to peak.

**Figure 2:**
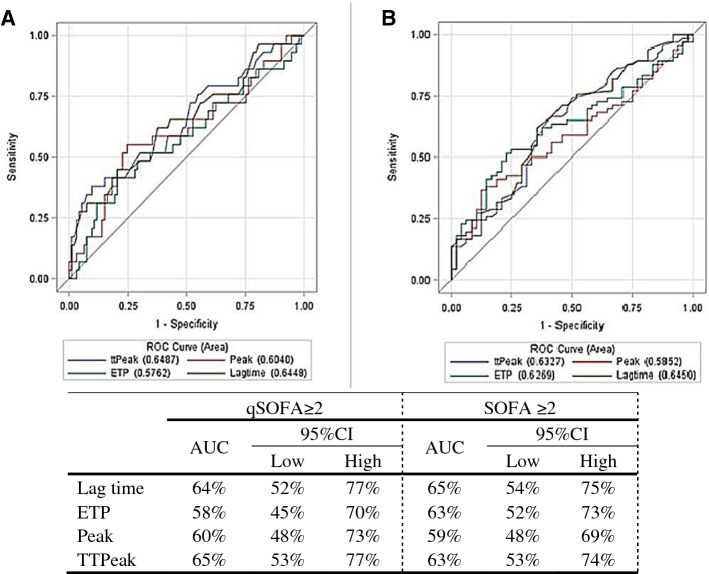
ROC curves in predicting high levels of qSOFA and SOFA. (**A**) represent the thrombin generation parameters in predicting high level of qSOFA (2–3 scores); (**B**) represent the thrombin generation parameters in predicting high level of SOFA (2 + scores). *ROC* receiver operating characteristic. *ETP* endogenous *thrombin* potential. *ttPeak* time to peak. *SOFA* sequential organ failure assessment. *qSOFA* quick sequential organ failure assessment. AUC, area under the curve; *95%CI* 95% confidence interval. *SOFA* sequential organ failure assessment.

With regard to infection severity, as determined by the SOFA score, patients with higher SOFA scores had a longer lag time (5.2 vs. 4.3 min), lower ETP value (1834 vs. 2015 nM*min) and longer TTpeak (7.5 vs. 6.7 min), with p = 0.008, 0.048, and 0.019, respectively (Table 2). After adjustment for risk factors and co-morbidities, lag time difference maintained statistical significance (Table 3). A positive correlation between lag time and SOFA (0.3) and TTpeak and SOFA (0.27) was noted, p = 0.001 and 0.004, respectively. A negative correlation was observed between ETP and SOFA (− 0.16), and peak height and SOFA (− 0.12), p = 0.09, 0.19 respectively (Table 4). The predictive abilities of TG parameters and the high qSOFA and SOFA scores were investigated using ROC curves and are presented in Fig. 2.

For qSOFA, the ROC curve showed an area under the curve (AUC) of 64% for lag time and 65% for TTpeak. For SOFA ≥ 2, the ROC curve showed an AUC of 65% for lag time, 63% for ETP and 63% for TTpeak.

Eleven patients died during hospitalization (9%). These patients had lower ETP (1,648 vs. 1,928 nM*min; p = 0.034) and peak height values (284 vs. 345 nM; p = 0.012), and a longer time-to-peak value (8.1 vs. 7.1 min; p = 0.052) than the survivors (Table 5).

**Table 5:** Association between thrombin generation and hospital mortality.

	Death		P-value
	No	Yes	
	N = 111	N = 11	
Lag time (min)	4.7 (1.5)	5.6 (2)	0.1046
ETP (nmol*min)	1928.8 (497.2)	1648.2 (292.6)	0.0349
Peak (nmol)	345 (70.2)	284.2 (76.1)	0.012
TTPeak (min)	7.1 (1.6)	8.1 (2)	0.0524

Variables are presented as the mean (SD).

*Min* minute, *ETP* endogenous thrombin potential, *TTPeak* time to peak.

**Figure 3:**
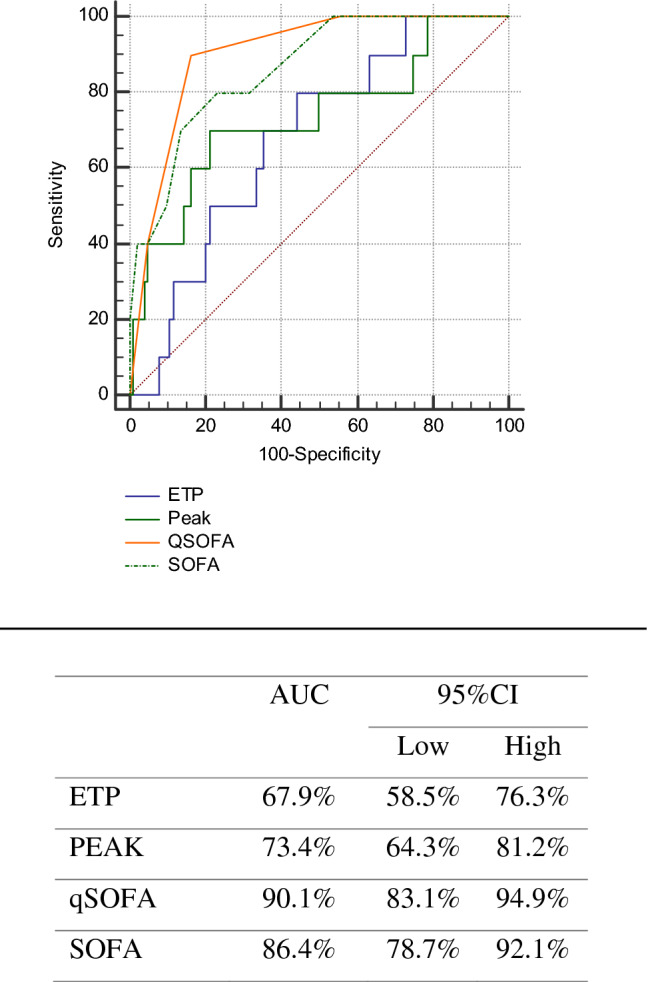
Under the ROC curve for the correlation of thrombin generation, qSOFA, SOFA and mortality. The diagnostic ability of thrombin generation values, qSOFA and SOFA in predicting mortality in sepsis. ROC, receiver operating characteristic. *AUC* area under curve. *CI* confidence interval. *qSOFA* quick sequential organ failure assessment. *SOFA* sequential organ failure assessment. *ETP* endogenous thrombin potential.

The ROC curves for TG and mortality showed an AUC of 67.9% for ETP, 73.4% for peak height, 90.1% for qSOFA and 86.4% for SOFA (Fig. 3). Combining the SOFA score and TG values did not result in a significantly better predictive ability, with an AUC of 0.868 for SOFA and ETP, and 0.878 for SOFA and peak height.

## Discussion

In our study, which involved patients with systemic infection, the results demonstrated that in both infection severity scores, qSOFA and SOFA, the clotting time values (lag time and TTpeak) were longer as infection severity increased. Additionally, the clotting time values exhibited relatively good discriminative abilities for infection severity with area under the ROC curve of approximately 65%. A trend of an inverse relationship between both peak height and ETP values with infection severity was noticed in both scores. Overall, we concluded that as infection severity increases, the potential ability for TG production in-vitro decreases. Although systemic infection in general, and sepsis specifically, are considered to be a hypercoagulative states, past research results are inconclusive. Similar to our results, some other studies have also correlated sepsis with a decreased potential ability to generate thrombin. Mihajlovic et al. demonstrated in 150 ICU patients that, as sepsis severity increased, ETP values decreased, however other TG values were not included in the trial^10^. Andersen et al. showed reduced TG values in 36 patients with sepsis and DIC compared with healthy controls^11^. Other, former observations have also shown reduced TG values in patients with sepsis in general and DIC in particular, yet these interesting studies did not include patients from the whole sepsis spectrum and compared values to healthy controls, not distributing according to sepsis severity^12–14^. Other studies published conflicting results^15–17^.

With regards to TG and mortality, patients with lower TG values had higher mortality rates. Most noticeable are the ETP and peak height values, which portray a good discriminatively ability. Consistent with our results, studies by Seo et al. and Massion et al. correlated low TG values with mortality in patients with sepsis^12,18^.

Thus, our findings strengthen past observations and, perhaps, resolve conflicting and confusing results, showing a correlation between increased infection severity and decreased potential ability to generate thrombin. Uniquely, our study evaluated infection severity by means of validated, routinely used scores, namely, SOFA and qSOFA, which their association with TG have not been examined thoroughly, if at all, to date. These results could have two possible explanations. As infection severity increases, a hypocoagulative state is formed. Or, conversely, and perhaps more reasonably, as infection severity increases extremely, it causes the consumption of clotting factors, which in turn, leads to low TG values.

These results imply that TG assays, particularly CAT, could be an effective and easily accessible tool in hospital settings to assess the severity and prognosis in these complex patients. Data concerning reference values is scarce and has been gathered only recently^19^, further studies are needed to determine reference values and to explore the possibility of TG assays in impacting and guiding the decision-making process in these complex patients and complex scenarios.

Our study had some limitations. Most of our patients had low qSOFA and SOFA scores. Perhaps the results would have been more conclusive if a greater number of patients had had higher score representations. The low mortality rate raises concern about the power of this patient population to draw conclusions. Moreover, we calculated qSOFA and SOFA after recruitment, therefore a formal diagnosis of sepsis could not have been done during recruitment. However, this enrollment allows for studying patients from the whole infection severity spectrum. Lastly, there is very little data regarding reference values for the CAT at the moment, thus we encourage further research in this field.

## Conclusion

TG assays may be a valuable tool in predicting infection severity and prognosis. Moreover, TG values suggest that, in severe infection, there is a reduced potential of generating thrombin.

## Data Availability

The datasets generated during and/or analyzed during the current study are available from the corresponding author on reasonable request.
